# Behavioral selection in structured populations

**DOI:** 10.1007/s12064-024-00413-8

**Published:** 2024-03-05

**Authors:** Matthias Borgstede

**Affiliations:** University of Bamberg, Markusplatz 3, 96047 Bamberg, Germany

**Keywords:** Selection by consequences, Behavioral selection, Natural selection, Price equation, Covariance based law of effect, Multilevel model of behavioral selection

## Abstract

The *multilevel model of behavioral selection* (MLBS) by Borgstede and Eggert (Behav Process 186:104370. 10.1016/j.beproc.2021.104370, 2021) provides a formal framework that integrates reinforcement learning with natural selection using an extended Price equation. However, the MLBS is so far only formulated for homogeneous populations, thereby excluding all sources of variation between individuals. This limitation is of primary theoretical concern because any application of the MLBS to real data requires to account for variation between individuals. In this paper, I extend the MLBS to account for inter-individual variation by dividing the population into homogeneous sub-populations and including class-specific reproductive values as weighting factors for an individual’s evolutionary fitness. The resulting formalism closes the gap between the theoretical underpinnings of behavioral selection and the application of the theory to empirical data, which naturally includes inter-individual variation. Furthermore, the extended MLBS is used to establish an explicit connection between the dynamics of learning and the maximization of individual fitness. These results expand the scope of the MLBS as a general theoretical framework for the quantitative analysis of learning and evolution.

## Introduction

Darwinian thinking has a long tradition in behavior analysis (e.g., Broadbent 1961; Campbell 1956; Gilbert 1970; Herrnstein 1964; Hull et al. 2001; Pringle 1951; Skinner 1966; Thorndike 1900). Although there seems to be a wide consensus that reinforcement learning can be described by analogy with natural selection, opinions about how exactly behavioral selection^1^ is connected to natural selection diverge. For example, Skinner (1981) claims that natural selection and reinforcement learning are two instances of the same underlying causal principle: selection by consequences. A different view is articulated by McDowell (2013), who proposes that learning and evolution constitute a single, self-similar process. Others have taken the position that the analogy between natural selection and behavioral selection is misleading because mechanisms of learning are themselves subject to natural selection (Burgos 2019).

The intricate relation between learning and evolution has also been acknowledged in evolutionary biology (e.g., Dunlap and Stephens 2016; McNamara and Houston 2009; Stephens 1991). Biological accounts of learning often focus on specific learning mechanisms as the target of natural selection (Aoki and Feldman 2014; Dridi and Lehmann 2014; Fawcett et al. 2013; Moore 2004). However, none of these approaches provides a general theoretical integration of learning and evolution by means of a formal model of selection. Such an integrated perspective would not only help to understand how evolution and learning interact, but also clarify under which conditions it is justified to assume that individual learning may lead to increases in individual fitness (cf. Frankenhuis et al. 2019; Lewis et al. 2010; Singh et al. 2010).

The multilevel model of behavioral selection (MLBS) aims at clarifying the relation between learning and evolution from the perspective of selection theory (Borgstede and Eggert 2021). The MLBS is a direct extension of the quantitative theory of natural selection as described by the Price equation (Price 1970, 1972). Therefore, all conclusions drawn from the model can be interpreted as mathematical theorems of natural selection. Following the rationale of multilevel selection theory (cf. Gardner 2015), selection is described both, between and within individuals.

The MLBS describes learning as an interaction between an individual and its environment on a molar level (i.e., it treats behavior as extended over time), thereby shifting the focus from molecular mechanisms like associative learning to statistical measures of behavioral allocation like mean, variance and covariance (cf. Baum 1973; Rachlin 1978). One of the core features of the MLBS is the use of statistical fitness predictors (“fitness proxies”) to define reinforcers, thereby establishing a functional relation between reinforcement learning and natural selection. In this view, a reinforcer is not a thing that is received by the individual, it is an event that co-varies with evolutionary fitness, given the conditions under which the organism has been shaped by natural selection. Such fitness predictors are phylogenetically important events (PIEs), as introduced by Baum (2012). A PIE that positively co-varies with evolutionary fitness will foster positive selection (i.e., reinforcement), whereas a PIE that negatively co-varies with fitness will result in negative selection (i.e., punishment). By replacing the concept of a reinforcer by the concept of PIEs, it is possible to analyze individual behavioral adaptations with regard to their evolutionary function, rather than the specific (molecular) mechanisms that realize this function.

The MLBS has inspired several theoretical papers that link the principle of behavioral selection to other analytical frameworks for behavior analysis, such as information seeking (Borgstede 2021) or matching theory (Borgstede and Luque 2021). Furthermore, first empirical applications of the framework have been proposed in Strand et al. (2022) and Borgstede and Anselme (2024).

However, the formalism introduced by Borgstede and Eggert (2021) builds on the simplifying assumption of a homogeneous population. From a strictly formal position, such a restriction would rule out all inter-individual variation apart from the selected behavior. Borgstede and Eggert (2021) argue that the assumption of a homogeneous population is justified because the theory deals with laws of behavior on the most general level. Moreover, it is possible to justify individual selection estimates as proposed in Borgstede and Anselme (2024) by stating the MLBS for an abstract population of individuals that are conceived to be identical to the individual under study. Although formally consistent, it is difficult to give a theoretically sound interpretation to such fictitious populations. A more elegant way to connect the empirical methodology by Borgstede and Anselme (2024) to the theoretical underpinnings presented in Borgstede and Eggert (2021), would be to include inter-individual variation in the formal presentation of the MLBS. Dropping the assumption of a homogeneous population would further widen the scope of the theory considerably, especially with regard to its evolutionary foundations. Therefore, in this paper, I extend the MLBS to account for inter-individual variation. The aim is to close the theoretical gap between the formalization of behavioral selection in the MLBS and possible empirical applications of the theory that naturally require to account for individual differences. The results will then be used to investigate the relation between individual learning dynamics and the idea that individual learning maximizes evolutionary fitness. While the latter is routinely assumed in many models of behavioral ecology (cf. Davies et al. 2012), it has never been shown on a theoretical level that fitness maximization models may in fact be applied to individual learning.

In the remainder of the paper, I will first review the core concepts and formalism of the MLBS as presented in Borgstede and Eggert (2021). I will then proceed to incorporate variation between individuals by dividing the population into homogeneous sub-populations, so-called “classes.” These classes formally account for individually different environments, constant individual traits, as well as regionally or culturally separated groups of individuals (Caswell 2001). Incorporating class-structure into the MLBS thus extends the scope of the model to arbitrary sources of inter-individual variation. The resulting generalized MLBS introduces the concept of *reproductive value* in the theory of behavioral selection. Reproductive value captures the relative contribution of a class of individuals to the future population and thus constitutes a weighting factor for the calculation of evolutionary fitness (Grafen 2006). The main result is that the principle of behavioral selection remains valid in inhomogeneous populations if individual fitness is evaluated in terms of expected gain in reproductive value. This result is further exploited to provide a formal justification for the assumption that individual learning tends to result in fitness maximization.

## The multilevel model of behavioral selection

The multilevel model of behavioral selection describes behavioral selection as an integral component of natural selection. The general idea is that behavioral change within individuals can be described by means of natural selection, if (a) individuals are treated as their own offspring and (b) evolutionary fitness is replaced by a statistical fitness predictor at the individual level. Evolutionary fitness is defined as the contribution of an individual to the future population (if individuals are treated as their own offspring, this corresponds to their survival probability).

Formally, the model relies on the most general description of natural selection as provided by the Price equation (Price 1970, 1972). The Price equation describes the average change of an evolving character from one generation (parents) to the next (offspring) by decomposing the change in mean character value $\Delta \overline{b }$ into one covariance term (selection) and one expectation term (non-selection)^2^:

1 $$\overline{w }\Delta \overline{b }={\text{Cov}}\left({w}_{i},{b}_{i}\right)+{\text{E}}({w}_{i} \Delta {b}_{i})$$

Here, ${b}_{i}$ is the character value of parent $i$ and ${w}_{i}$ designates the contribution of parent $i$ to the offspring generation (i.e., the relative frequency of individuals in the offspring generation that descend from parent $i$). It is important to note that the change in mean character value $\Delta \overline{b }={{\text{E}}(b}_{i}{\prime}-{b}_{i})$ is defined with respect to the parent generation, as well. This means that ${b}_{i}{\prime}$ refers to the mean character value of parent $i$’s offspring (i.e., the index always refers to the parent). $\overline{w }$ is defined as the average contribution of parents to the offspring generation. With these definitions, the Price equation is a mathematical truth. In other words, for any two sets that can be related in the sense of “parents” and “offspring,” the change in mean character value corresponds to the covariance between character value and individual fitness plus the expectation over the difference between a parents’ and their offspring’s character values. This mathematical insight describes the concept of change by a process of selection on the most general level. The Price equation is true for arbitrary characters and for arbitrary mechanisms of inheritance. Therefore, it seems plausible that it also provides a suitable mathematical background for the description of reinforcement learning (cf. Price 1995, written ca. 1971).

The potential of the Price equation as a formal account of reinforcement learning was further explored by Baum (2017). Baum’s formal account of behavioral selection using the Price equation builds on the idea that *behaviors* are the elementary units of selection. Here, selection refers to the change in average behavioral allocation between one set of reinforcement trials (the “parent population”) and a later set of reinforcement trials (the “offspring population”). While providing a mathematically sound description of behavioral change for some special cases, Baum’s account does not provide a complete account of how “parent behaviors” produce “offspring behaviors,” hence it is difficult to make sense of the corresponding behavioral fitness equivalent (Borgstede and Eggert 2021).

The MLBS takes a slightly different route, treating individuals as the elementary units of selection. Individuals behave, individuals reproduce and individuals die, whereas behaviors do nothing of the above. Therefore, fitness is defined at the individual level as the contribution of an individual to the future population (i.e., using the standard definition of evolutionary fitness as described above). Second, behavioral selection occurs within individuals. This means that the effect of natural selection as captured by the covariance term in the Price equation is negligible for behavioral selection. Therefore, the MLBS focuses on the expectation term, restricting the analysis to the survival part of evolutionary fitness. Third, because fitness is an individual characteristic, there is no variation in fitness within individuals (and hence no covariance between fitness and any target of behavioral selection). In order to enable selection, the individual is therefore assumed to adapt its behavior to the environment using statistical fitness proxies.

Adopting a molar view on behavior, the MLBS describes the change in mean behavioral allocation, where behavioral allocation is itself taken to be extended over time. For example, one may express the behavioral allocation of a foraging animal as the relative time spent at each food patch within a certain interval. These intervals constitute *behavioral episodes* and are defined by a uniform class of recurring contextual factors (i.e., by a certain structure of reinforcement contingencies). Behavioral change by means of selection is thus specified on two different levels, the level of mean population behavior (averaged over individuals) and the level of mean individual behavior (averaged over behavioral episodes) with behavioral episodes being nested within individuals.

Building on these concepts, it is now possible to describe the within individual change ${w}_{i}\Delta {b}_{i}$ within the expectation term of the Price equation by recursive expansion (i.e., by inserting the Price equation into itself). To indicate the different levels of selection, I introduce the index $i$ for expected values and covariances at the population level and the index $j$ for expected values and covariances at the within-individuals level:

2 $$\overline{w }\Delta \overline{b }={{\text{Cov}}}_{i}\left({w}_{i},{b}_{i}\right)+{{\text{E}}}_{i}\left({{\text{Cov}}}_{j}\left({w}_{ij},{b}_{ij}\right)+{{\text{E}}}_{j}\left({w}_{ij}\Delta {b}_{ij}\right)\right)$$

Consequently, the fitness-weighted change within individuals is given by:

3 $${w}_{i}\Delta {b}_{i}={{\text{Cov}}}_{j}\left({w}_{ij},{b}_{ij}\right)+{{\text{E}}}_{j}\left({w}_{ij}\Delta {b}_{ij}\right)$$

Equation 3 describes behavioral selection within individual $i$ as the within individual covariance between behavioral allocations over episodes $j$ (${b}_{ij}$) and the corresponding evolutionary fitness ${w}_{ij}$. The second term refers to any changes in behavioral allocation within individual $i$ apart from the selection component. In the MLBS, the varying fitness values ${w}_{ij}$ are further replaced by the predicted values from a linear regression of the form ${{{w}_{ij}=\beta }_{0}+\beta }_{wp}{p}_{ij}+\varepsilon$. Note that the regression coefficients refer to the population level here. Inserting this in the above equation (and defining ${{\text{E}}}_{j}\left({w}_{ij}\Delta {b}_{ij}\right)=\delta$) yields:

4 $${w}_{i}\Delta {b}_{i}={{\text{Cov}}}_{j}\left({{\beta }_{0}+\beta }_{wp}{p}_{ij},{b}_{ij}\right)+\delta$$

which can be rearranged to:

5 $${w}_{i}\Delta {b}_{i}={\beta }_{wp}{{\text{Cov}}}_{j}\left({p}_{ij},{b}_{ij}\right)+\delta$$

Equation 5 is called the *covariance based law of effect* (CLOE) since it describes the expected change in mean behavioral allocation due to behavioral selection in a quantitative way. The term ${\beta }_{wp}$ refers to the regression slope of evolutionary fitness on $p$ and is called the”reinforcing power” of a fitness proxy. Thus, mean change in behavioral allocation is proportional to the covariance between behavior and a statistical fitness predictor with the reinforcing power of the predictor as a scaling factor.

By linking the concept of behavioral selection to the theory of natural selection, the MLBS gives a quantitative account of reinforcement at the most general level. It gives a valid description of all processes of behavioral selection regardless of the specific mechanisms involved. However, in its current form, the model makes one limiting assumption: individuals are treated as if they were identical when conditioned on $p$. This means that strictly speaking, the CLOE is only valid if all fitness predictors apart from $p$ are distributed randomly over the individuals. This is a considerable limitation of the scope of the theory and shall thus be addressed in the following section.

## Introducing variation to the MLBS

In biological models of natural selection, inter-individual variation is usually captured by dividing the population into homogeneous sub-populations, so called *classes* (e.g., Batty et al. 2014; Grafen 2020; Taylor 1990). These classes may refer to different stages in the life cycle of the species (e.g., juvenile, adult, post-reproductive…), different sexes (male, female, hermaphrodite…), age (e.g., in seasonal species), spatial distribution (e.g., different food patches) or any other personal or environmental characteristic that is relevant for evolutionary fitness (Caswell 2001). The idea of introducing a class-structure is to ensure that, within classes, individuals are identical with respect to any characteristics affecting their evolutionary fitness. This means that any variation that is not captured by the class-structure is assumed negligible with regard to evolutionary fitness.

There are different versions of the Price equation for class-structured populations with various notations, depending on the type of class-structure and the aim of the underlying model (e.g., Grafen 2015; Lion 2018; Taylor 1990). Nevertheless, these different formulations all follow the same general rationale. First, natural selection is formulated by means of the Price equation for each class separately. And second, the overall population change of the evolving character is calculated as a weighted mean of the contributions from each class. Formally, for the Price equation to make sense, the weighting factors are arbitrary. However, in the context of biological evolution, adequate weighting factors have been shown to be unique only up to a positive constant and correspond to the *reproductive values* of the corresponding classes (Batty et al. 2014; Grafen 2006, 2015; Taylor 1990). Reproductive values were introduced by Fisher (1930) and refer to the relative genetic contribution of an individual to the future population. As such, reproductive values depend on class-specific fertility rates and reproduction rates (i.e., they are demographic parameters). Given a demographic model of the population dynamics, reproductive values can be calculated using standard methods from matrix algebra (Caswell 2001, 2010). For reasons of mathematical convenience, in the following I assume reproductive values to be scaled such that the sum of all class reproductive values ${v}_{x}$ is one. The weighted average thus reduces to a weighted sum over classes.

The Price equation for classes can now be stated as:

6 $$\overline{w }\sum_{x}{v}_{x}{\Delta \overline{b} }_{x}=\sum_{x}{v}_{x}{{\text{Cov}}}_{x}\left({w}_{i},{b}_{i}\right)+\sum_{x}{v}_{x}{{\text{E}}}_{x}\left({w}_{i}\Delta {b}_{i}\right)$$

Note that covariances and expectation values are taken not over all individuals, but conditional on the respective classes $x$. Moreover, the “average change” in population value $\Delta \overline{b }$ in the class-structured case is also actually a weighted average of the conditional mean changes for each class (${\Delta \overline{b} }_{x})$ with the class reproductive values (${v}_{x}$) as weighting factors. Finally, individual fitness ${w}_{i}$ is defined as a weighted sum over the number of offspring ${n}_{y}$ in each class $y$ multiplied by the corresponding offspring reproductive values ${v}_{y}$:

7 $${w}_{i}=\sum_{y}{v}_{y}{n}_{iy}$$

Like in the simple case without classes, it is straightforward to expand the expectation term by applying the Price equation recursively to describe mean character change from individual $i$ to the descendants of individual $i$, i.e., ${w}_{i}\Delta {b}_{i}$. Since the MLBS focuses only on the survival part of natural selection, this corresponds to within individuals change in behavioral allocation ${b}_{i}$ from one set of behavioral episodes to the next. Furthermore, as long as individuals only change classes between these time steps, all behavioral episodes $j$ within individuals $i$ correspond to the same class $x$. Therefore, the class subscript can be omitted and the equation for behavioral selection remains unchanged^3^:

8 $${w}_{i}\Delta {b}_{i}={{\text{Cov}}}_{j}\left({w}_{ij},{b}_{ij}\right)+{{\text{E}}}_{j}\left({w}_{ij}\Delta {b}_{ij}\right)$$

Like in the simple MLBS without class-structure, behavioral selection is expressed with respect to a statistical fitness predictor $p$, rather than fitness itself. However, since individual fitness is now defined using offspring reproductive values ${v}_{y}$ as weighting factors, it is necessary to specify the effect of $p$ with respect to each possible subsequent class $y$. This means that instead of one simple linear regression of fitness on $p$, we now use a linear regression of each fitness component on $p$. These fitness components correspond to the offspring classes, i.e., the possible classes $y$ to which the individual may transition from the current class $x$. Formally, this is accomplished by a class specific linear regression for each number ${n}_{y}$ of descendants in offspring class $y$ of the form: ${n}_{y}={\beta }_{xy0}+{\beta }_{xyp}p+\varepsilon$. Like in the simple case, these regressions are defined on the level of the population. The evolutionary fitness of individual $i$ can thus be written as:

9 $${w}_{i}=\sum_{y}{v}_{y}\left({\beta }_{xy0}+{\beta }_{xyp}p+\varepsilon \right)=\sum_{y}{v}_{y}{\beta }_{xy0}+{{v}_{y}\beta }_{xyp}p+{v}_{y}\varepsilon$$

Substituting this for the predicted fitness in the behavioral selection part of the Price equation yields:

10 $$\begin{aligned}{w}_{i}\Delta {b}_{i}={{\text{Cov}}}_{j}\left(\sum_{y}{v}_{y}{\beta }_{xy0}+{{v}_{y}\beta }_{xyp}{p}_{ij},{b}_{ij}\right)+\\{{\text{E}}}_{j}\left(\sum_{y}{v}_{y}{\beta }_{xy0}+{{v}_{y}\beta }_{xyp}{p}_{ij}\Delta {b}_{ij}\right)\end{aligned}$$

Since we are concerned with the selection part only, we simplify by treating the expectation term as a residual $\updelta$. Within-individuals change in behavioral allocation can now be rearranged to:

11 $${w}_{i}\Delta {b}_{i}=\sum_{y}{v}_{y}{\beta }_{xyp}{{\text{Cov}}}_{j}\left({p}_{ij},{b}_{ij}\right)+\delta$$

This is the covariance based law of effect with class-structure. Its general form resembles the simple version derived in Borgstede and Eggert (2021): behavioral selection is proportional to the covariance between behavioral allocation and an arbitrary fitness predictor. However, in the class-structured case, the reinforcing power of $p$ is now defined as the reproductive value weighted sum over the effects of *p* on all fitness components $y$ (i.e., $\sum_{y}{v}_{y}{\beta }_{xyp}$).

## Learning and fitness maximization

Several authors have approached individual learning from a maximization perspective, where stable state behavior is analyzed in terms of maximal reinforcer value (cf. Rachlin et al. 1981, 1976; Rachlin and Burkhard 1978). Reinforcer value in this context is closely connected to the concept of subjective utility from behavioral economics (Herrnstein et al. 1993; Loewenstein et al. 2009). Similarly, behavioral ecology routinely applies fitness maximization as an explanatory mode for behavioral adaptations (cf. Caswell 1982; Davies et al. 2012). Although the latter is concerned with evolutionary adaptations (i.e., the possible outcomes of natural selection), many models in behavioral ecology actually refer to the outcome of individual level adaptations. For example, the ideal free distribution (IFD) describes how individuals in a population are distributed over two different food patches when the outcome is negatively related to the number of individuals that already are at the patch. Taking consumed food as a proxy of evolutionary fitness, individual fitness will be maximal for each individual if and only if the ratio of individuals at the two patches matches the ratio of food resources at the two sites (Fretwell 1972; Fretwell and Lucas 1969). Although the formal model is meant to explain evolutionary adaptations, the actual distribution of individuals over food patches is hardly ever the result of natural selection. The model correctly predicts that natural selection should produce an IDF for plants or other organisms whose spatial position is fixed over the lifetime of a single individual. However, many applications of the IFD involve moving animals, whose spatial position with regard to food patch distribution is often not the result of natural selection, but rather of individual learning (Houston 2008; Kraft et al. 2002). To apply an evolutionary model of fitness optimization to individual behavioral adaptations (i.e., learning), presupposes that learning can be explained by the same principles as evolution. Many results from behavioral ecology seem to imply that this might indeed be the case (cf. Davies et al. 2012; Stephens and Krebs 1986). However, unless evolutionary theory and learning theory are described within a unified model, the assumption that individual learning leads to the maximization of evolutionary fitness is unjustified.

The MLBS may provide such a unified theoretical perspective and thus close the theoretical gap between individual learning and fitness maximization. In the following, I will establish an explicit link between the class-structured version of the CLOE developed above and the principle of fitness maximization by exploiting the formal equivalence of evolutionary fitness and reinforcer value established in Borgstede (2020). The paper argues that if there is a maximand of reinforcement learning, it must be defined such that maximizing reinforcement coincides with maximizing evolutionary fitness. The link between reinforcement and evolutionary fitness is then established under the assumption that maximization occurs on both levels.

However, the theoretical framework in Borgstede (2020) does not include the dynamics of learning (as described by the CLOE), but only the possible endpoints of individual learning given that learning maximizes reinforcer value. Inter-individual variation is modeled by an arbitrary class-structure, with evolutionary fitness being the reproductive value weighted sum of an individual’s offspring. Like in this paper, individuals are formally treated as their own offspring, thereby bridging the different time scales of natural selection and behavioral selection. The main difference is that the notation is slightly different in Borgstede (2020), using partial derivatives to define the key concepts. These partial derivatives correspond to the partial regression coefficients used to derive the CLOE. Therefore, the marginal change in fitness components $y$ per unit change in behavior (designated ${\pi }_{xy}({b}_{x})$ in Borgstede 2020) corresponds to the partial regression coefficients ${\beta }_{xyp}$ introduced above. Thus, the definition of “reinforcing power” in the maximization paper is identical to the one derived above and corresponds to the reproductive value weighted sum of fitness effects (i.e., $\sum_{y}{v}_{y}{\beta }_{xyp}$). Marginal reinforcer value of a behavior $b$ in class $x$ is further designated $r\left({b}_{x}\right)$ and defined as the product of the reinforcing power of $p$ and the marginal change in $p$ per unit change in behavioral allocation. Like before, this marginal change (designated ${p}_{x}$ in Borgstede 2020) can be identified with the slope of a linear regression of the form ${p}_{ij}={\beta }_{x0}+{\beta }_{xpb}{b}_{ij}+\varepsilon$. To retrieve the slope ${\beta }_{xpb}$ from the CLOE, one can write the covariance between behavior and $p$ as:

12 $${{\text{Cov}}}_{j}\left({p}_{ij},{b}_{ij}\right)={\beta }_{xpb}{{\text{Var}}}_{j}({b}_{ij})$$

Following the definition in Borgstede (2020), the marginal reinforcer value of mean behavioral allocation of an individual in class $x$ can be re-stated as:

13 $$r\left({b}_{x}\right)={\beta }_{xpb}\sum_{y}{v}_{y}{\beta }_{xyp}$$

If an individual maximizes this value, it also maximizes its evolutionary fitness (Borgstede 2020). We can further rearrange the CLOE to get:

14 $${w}_{i}\Delta {b}_{i}={\beta }_{xpb}\sum_{y}{v}_{y}{\beta }_{xyp}{{\text{Var}}}_{j}\left({b}_{ij}\right)+\delta$$

which is equivalent to:

15 $${w}_{i}\Delta {b}_{i}=r\left({b}_{i}\right){{\text{Var}}}_{j}\left({b}_{ij}\right)+\delta$$

Thus, in the MLBS, behavioral selection equals the product of marginal reinforcer value and (within-individuals) behavioral variance. This means that, if there is no variation in behavior, there is no behavioral selection. Furthermore, behavioral selection only occurs if changes in behavior are associated with changes in reinforcer value, and thus, evolutionary fitness. Consequently, if a behavior is optimal with regard to evolutionary fitness (such as the distribution of individuals over food patches according to the IFD), the MLBS predicts that this behavior will also be selected by individual learning as described by the CLOE. This result integrates individual learning dynamics with the concept of fitness maximization via the abstract principle of behavioral selection.

## Conclusion

This paper presents an extension of the multilevel model of behavioral selection (MLBS) to account for inter-individual variation. Variation between individuals is modeled by dividing the population into homogeneous classes. These classes can represent any source of variation between individuals, be it internal or external. Within this framework, a generalized version of the covariance based law of effect (CLOE) was derived. It turned out that the CLOE remains valid even if there is variation between individuals. The only difference to the simple version without classes is that the reinforcing power of a fitness predictor is now defined as a reproductive value weighted sum of fitness effects. This general result was then exploited to close a theoretical gap between evolutionary models of fitness maximization and individual learning dynamics*.* By linking the theory of behavioral selection to the concept of reproductive value, fitness maximization turns out to be a natural result of individual behavioral adaptations when learning is understood as a selection process as described in the MLBS.

The main implications of the extended MLBS for class-structured populations are thus that (a) applications of behavioral selection models to individuals from an inhomogeneous population are valid and (b) applications of evolutionary maximization models to the outcomes of individual learning are valid. Both results have a high intuitive appeal, which is probably why they have hardly been questioned in the past. Although the formal justification of these two assumptions has no practical implications, it is of great theoretical value to know that the current modus operandi is indeed theoretically sound.

The formalism presented here is not the first to introduce inter-individual variation into a multilevel Price equation. Gardner (2015) incorporates class-structure into a multilevel Price equation that bears some similarity to the one derived here. Although concerned with group-level and individual-level selection (rather than between-individuals and within-individuals selection as the MLBS), the two approaches address a similar problem. Nevertheless, the MLBS partitioning substantially differs from Gardner's derivation both technically and conceptually. On a technical level, Gardner uses parental reproductive value as the target of selection, thereby allocating class transitions in the offspring generation. Conceptually, this implies that Gardner's partitioning exclusively focuses on selection due to reproduction. However, since the MLBS is concerned with learning (i.e., selection within individuals), the relevant aspect of selection is not reproduction, but survival (i.e., selection due to class transitions). Therefore, although Gardner's formalism is perfectly adequate as a basis of a genetical theory of selection, it does not account for selection that may occur within individuals. In contrast to Gardner’s approach, the MLBS, treats parental fitness as the target of selection, which is defined such that offspring are weighted according to the corresponding *offspring* reproductive values. Because a surviving individual may formally be treated as its own offspring, the MLBS accounts for fitness effects due to class transitions, which is a necessary condition for within-individuals selection.

The MLBS aims to provide a general explanatory framework for individual learning in an evolutionary context. It is not, however, intended to be a specific learning model for, say, associative or non-associative processes in a given species. As argued in Borgstede and Luque (2021), the MLBS is best understood as a conceptual framework for the quantitative analysis of behavior from an evolutionary perspective. Within this framework, the CLOE provides the fundamental principle of behavioral selection theory. Like the simple Price equation, given the definitions of the model primitives, the CLOE states a mathematical truth. Any empirical application would require to further specify the constraining conditions and auxiliary laws that are needed to describe a specific learning scenario (cf. Borgstede and Eggert 2023a,b). Given such specializations of the fundamental theoretical principles, the MLBS can indeed provide explanations for various well-known empirical phenomena, such as conditions of undermatching and overmatching in operant choice (Davison and McCarthy 2016) or the blocking of uninformative stimuli in classical conditioning (Kamin 1969), as shown in Borgstede and Eggert(2021) and Borgstede and Luque (2021). Further theoretical developments may extend the MLBS such that it could also account for adaptive behavior that is based on imitation or instruction. Such work may hopefully shed light on the connection between learning, evolution and culture.

Behavioral selection theory is only at the beginning of its development as a meta-theory of adaptive behavior. Instead of describing how associations are established within an individual by singular events, behavioral selection describes behavior on a larger time scale and is concerned with the long-term interactions between learning individuals and their environment. The MLBS thus provides a general formal framework to derive quantitative laws on a molar level, thereby linking the dynamics of learning to the theory of evolution by natural selection.

## Footnotes

1 In this paper, the term “behavioral selection” exclusively refers to learning processes on the level of the individual organism.

2 Note that the notation used for the Price equation varies considerably between publications. If the evolving character is an allele frequency, it is often designated $x$, $y$ or $z$. Sometimes authors differentiate between the phenotypic character and the part of it that is subject to evolutionary change, which is then referred to as either $g$ or $p$. Since in this paper I am concerned with behavior (as opposed to genetic values), I prefer the letter $b$.

3 An analogous result is presented by Gardner (2015) who argues that social groups may be considered as units of selection if the groups are homogenous with respect to their respective classes.
